# 3D Printed Monolithic Microreactors for Real-Time Detection of *Klebsiella pneumoniae* and the Resistance Gene *bla*_NDM-1_ by Recombinase Polymerase Amplification

**DOI:** 10.3390/mi11060595

**Published:** 2020-06-17

**Authors:** Ole Behrmann, Matthias Hügle, Franz Eckardt, Iris Bachmann, Cecilia Heller, Marina Schramm, Carrie Turner, Frank T. Hufert, Gregory Dame

**Affiliations:** 1 Institute of Microbiology and Virology, Brandenburg Medical School Theodor Fontane, 16816 Neuruppin, Germany; ole.behrmann@mhb-fontane.de (O.B.); matthias.huegle@mhb-fontane.de (M.H.); franz.eckardt@b-tu.de (F.E.); iris.bachmann@mhb-fontane.de (I.B.); cecilia.heller@mhb-fontane.de (C.H.); marina.schramm@mhb-fontane.de (M.S.); frank.hufert@mhb-fontane.de (F.T.H.); 2 Laboratory for Sensors, Department of Microsystems Engineering - IMTEK, University of Freiburg, 79110 Freiburg, Germany; 3 National Infections Service, Public Health England, Porton Down SP4 0JG, UK; carrie.turner@phe.gov.uk

**Keywords:** recombinase polymerase amplification (RPA), antibiotic resistance, 3D printing

## Abstract

We investigate the compatibility of three 3D printing materials towards real-time recombinase polymerase amplification (rtRPA). Both the general ability of the rtRPA reaction to occur while in contact with the cured 3D printing materials as well as the residual autofluorescence and fluorescence drift in dependence on post curing of the materials is characterized. We 3D printed monolithic rtRPA microreactors and subjected the devices to different post curing protocols. Residual autofluorescence and drift, as well as rtRPA kinetics, were then measured in a custom-made mobile temperature-controlled fluorescence reader (mTFR). Furthermore, we investigated the effects of storage on the devices over a 30-day period. Finally, we present the single- and duplex rtRPA detection of both the organism-specific *Klebsiella* haemolysin (*khe)* gene and the New Delhi metallo-β-lactamase 1 (*bla*_NDM-1_) gene from *Klebsiella pneumoniae*. Results: No combination of 3D printing resin and post curing protocol completely inhibited the rtRPA reaction. The autofluorescence and fluorescence drift measured were found to be highly dependent on printing material and wavelength. Storage had the effect of decreasing the autofluorescence of the investigated materials. Both *khe* and *bla*_NDM-1_ were successfully detected by single- and duplex-rtRPA inside monolithic rtRPA microreactors printed from NextDent Ortho Clear (NXOC). The reaction kinetics were found to be close to those observed for rtRPA performed in a microcentrifuge tube without the need for mixing during amplification. Singleplex assays for both *khe* and *bla*_NDM-1_ achieved a limit of detection of 2.5 × 10^1^ DNA copies while the duplex assay achieved 2.5 × 10^1^ DNA copies for *khe* and 2.5 × 10^2^ DNA copies for *bla*_NDM-1_. Impact: We expand on the state of the art by demonstrating a technology that can manufacture monolithic microfluidic devices that are readily suitable for rtRPA. The devices exhibit very low autofluorescence and fluorescence drift and are compatible with RPA chemistry without the need for any surface pre-treatment such as blocking with, e.g., BSA or PEG.

## 1. Introduction

*Klebsiella pneumoniae* (KPN) is a Gram-negative bacterium and opportunistic pathogen belonging to the family Enterobacteriaceae. It is a natural colonizer of a multitude of body sites such as the gut, skin, respiratory tract, oropharynx, and the reproductive organs. Especially in immunocompromised patients, such as neonates [1] and the elderly, it is a major cause of difficult to treat drug-resistant urinary tract infections (UTIs), infections of soft tissue and open wounds, pneumonia, and sepsis. Naturally, KPN is resistant to penicillin, mediated by the chromosomal SHV-1 penicillase, but is able to greatly expand its antimicrobial resistance (AMR) profile by horizontal gene transfer (HTR). As such it is part of the “ESKAPE” group (***E****nterococcus faecium, **S**taphylococcus aureus, **K**lebsiella pneumoniae, **A**cinetobacter baumannii, **P**seudomonas aeruginosa, **E**nterobacter*) of pathogens which is a major driver of the current AMR crisis. Due to its high rate of HTR, mostly mediated by plasmids, many of the new AMR genes discovered in the last decades have been first isolated from KPN before spreading to other Gram-negative organisms. Overall, hundreds of mobile resistance genes are known to be associated with KPN [2]. Based on these facts, both the World Health Organization [3] as well as the European Union [4] consider KPN to be a major threat to public health.

First isolated from KPN carried by a patient of Indian origin in Sweden in 2008 [5], New Delhi metallo-β-lactamase-1 (NDM-1) confers resistance to almost all β-lactam antibiotics including carbapenems. Infections with NDM-1 expressing bacteria are thus only treatable with antibiotics of last-resort such as colistin. Natural reservoirs of *bla*_NDM-1_ have been found worldwide with a focus on the Indian subcontinent [6].

To prevent the spread of *bla*_NDM-1_ carrying KPN in healthcare settings, rapid screening methods are needed that accurately identify both the organism and AMR genes with high sensitivity. Today’s gold standard is the quantitative polymerase chain reaction (qPCR) that delivers results within about one hour [7] but is constrained to large laboratories due to the need for technically complex thermocyclers with built-in fluorescence detection equipment.

A novel isothermal alternative to (q)PCR is recombinase polymerase amplification (RPA) [8]. Here, the DNA double helix is opened enzymatically instead of by heat and annealed primers are extended by a DNA polymerase active at 39–42 °C. Amplification is complete within 5–15 min and, with the addition of a fluorescent probe [9], can be turned into a real-time format. Due to its availability as a ready to use lyophilized enzyme mix, simple incubation requirements as well as the ability to detect target sequences from crude lysates [10], RPA is an ideal candidate for the development of small and inexpensive nucleic acid detection equipment for use at the point of care. Up until now, a multitude of RPA based detection assays targeting bacteria [11,12,13,14], viruses [15,16,17,18,19,20], parasites [21,22,23], and microRNAs [24] have been developed. With the addition of a second primer pair and a corresponding fluorescent probe, RPA assays are also easily duplexed in a single reaction vessel [12,13].

RPA has been shown to work while in contact with a multitude of polymers commonly used to construct microfluidic systems such as cyclic olefin polymer (COP) [25], (poly)methyl methacrylate (PMMA) [26], polydimethylsiloxane (PDMS) [27], and acrylonitrile butadiene styrene (ABS) [28]. However, all of the aforementioned polymers were structured using classical microfabrication methods. Only very limited data is available for the compatibility of common 3D printing materials with RPA. Yamanaka et al. [29] performed RPA in open top microwell plates printed from black polylactic acid (PLA) by fused deposition modeling (FDM) which were sealed by either an overlay of mineral oil or adhesive foil. End-point readout of the reactions was then performed by hybridization of the amplicons to probes immobilized on a second polycarbonate chip followed by colorimetric detection.

When expanding to fluorescent real time detection of the RPA reaction, autofluorescence as well as fluorescence drift of the material of the reaction vessel become major parameters. Polymers commonly used for the construction of microfluidic systems have intrinsic autofluorescence that decreases due to bleaching under continuous illumination [30]. A recent study [31] of common materials used for FDM found that only a few brands of deep black ABS and PLA showed somewhat acceptable fluorescence behavior in a limited wavelength range from 300–400 nm, which falls outside of the spectra of the most commonly used fluorescent labels used for nucleic acid detection, such as those based on fluorescein. These findings show that FDM, unless new materials are developed, is unsuitable for the construction of monolithic microfluidic systems as the manufacture of optically clear parts with low autofluorescence and fluorescence drift is not possible. For parts manufactured by stereolithography (SLA), more promising results have been achieved. Kadimisetty et al. [32] have demonstrated successful real-time detection of loop mediated isothermal amplification (LAMP) assays in semi-monolithic micro reactors printed from clear resin (FLGPCL02, Formlabs, Somerville, MA, USA). However, surface coating with polyethylene glycol (PEG) or poly(vinyl alcohol) (PVA) was necessary to achieve satisfactory assay sensitivity for the detection of genomic DNA targets.

In this work we seek to advance the state-of-the-art by investigating whether it is possible to manufacture monolithic microreactors suitable for rtRPA by 3D printing technology based on digital light processing (DLP). Our goal is to find a combination of 3D printing material and a post curing protocol that meets the following criteria:

1. Optically clear
2. Non-inhibitory to RPA chemistry without the need for surface pre-treatment
3. Low autofluorescence
4. Low fluorescence drift

The basic formulation of a printing resin suitable for DLP based printing contains a monomer, a photoinitiator [33] and a blocking agent that limits penetration depth during curing [34]. Any of these three components individually or in combination may be responsible for autofluorescence depending on printing and post curing conditions.

First, we investigate monolithic microreactors printed from three methacrylate-based resins for autofluorescence and autofluorescence drift in dependence on post curing and storage conditions by means of a custom made heater/fluorescence detector. Finally, we demonstrate highly sensitive detection of both the *khe* and *bla*_NDM-1_ genes by rtRPA. 

## 2. Experimental

### 2.1. 3D Printed Monolithic Microreactors

**Figure 1:**
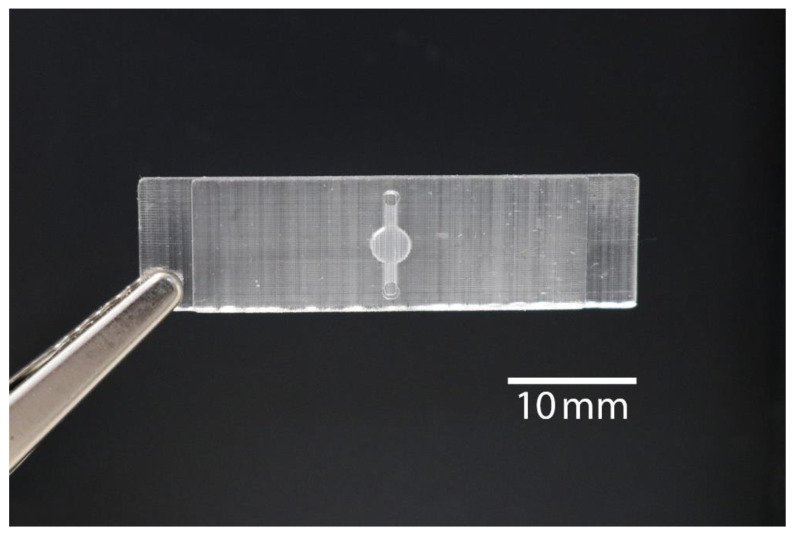
3D printed monolithic microfluidic device with buried real-time recombinase polymerase amplification (rtRPA) microreactor.

**Figure 2:**
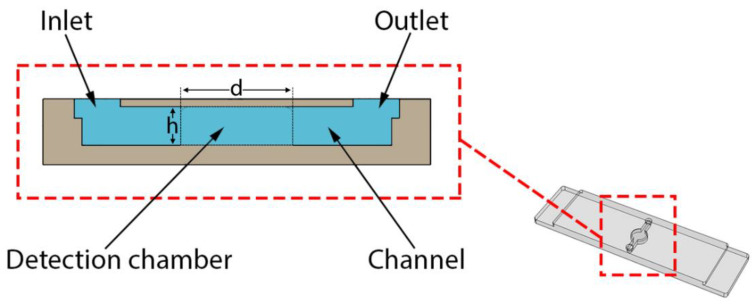
Cross-section of the 3D printed microfluidic device. The diameter (d) and height (h) of the detection chamber are 3 mm and 0.8 mm, respectively.

To perform rtRPA, a simple to operate single-use microfluidic device was developed (see Figure 1) and manufactured by DLP 3D printing. This approach allows the device to be manufactured in a single piece obviating the need to first manufacture a half shell that is subsequently sealed. The main component is a buried microreactor (diameter: 3 mm, height: 0.8 mm) connected to two ports for filling/emptying (diameter: 0.8 mm, height: 0.8 mm) by micropipette. The total volume is about 12 µL. The overall dimensions of the device are 38 mm × 10 mm × 1.7 mm. The thickness of the top lid covering the microreactor is 200 µm (see cross-section in Figure 2).

Devices were designed by computer aided design (CAD) (SolidWorks, Dassault Systèmes, Vélizy-Villacoublay, France) and subsequently exported to a 3D printing pre-processor/slicer (NetFabb, Autodesk, San Rafael, CA, USA). Here, support structures were added and slicing was performed at a layer thickness of 100 µm using settings specific to each of the tested printing materials (see Table 1). 3D printing was then carried out on a SolFlex SF350 (Way2Production, Vienna, Austria) DLP printer using the PowerVat printing bed.

After printing, remaining liquid printing material was let to drip back into the printing bed for at least 15 min. The parts were then removed from the printing platform and sonicated for 15 min in isopropyl alcohol (IPA) to remove remaining liquid material sticking to the surfaces. The microreactors were then thoroughly flushed with IPA using a wash bottle. Subsequently, the devices were carefully dried by compressed air and incubated at 40 °C for at least one hour to evaporate the remaining IPA. Finally, post curing was carried out according to the protocols detailed in the following section.

### 2.2. Post Curing

Post curing was performed by placing cleaned and air-dried parts into the FormCure (Formlabs, USA) post curing instrument. The FormCure uses high power LEDs at a center wavelength of 405 nm to illuminate parts placed on a central turntable and maintains a preset curing temperature from ambient temperature up to 80 °C. After curing, parts were stored protected from light at ambient conditions. Table 2 lists the details of the post curing protocols used in this work.

**Figure 3:**
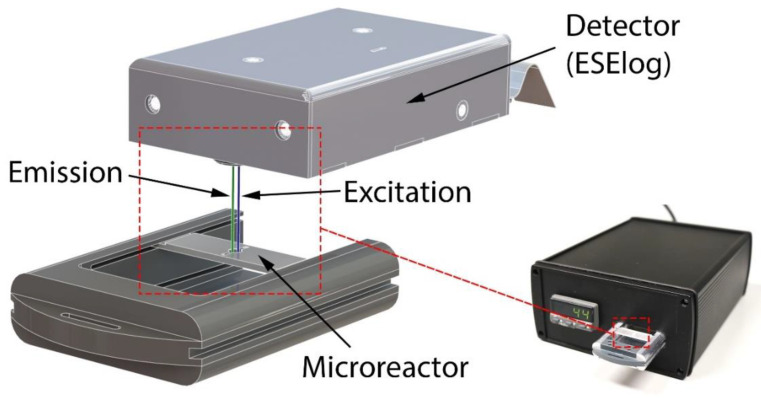
Mobile temperature-controlled fluorescence reader (mTFR) instrument. The 3D printed microreactor is placed in a carrier that is inserted into the instrument. Incubation temperature is set by the front panel controls on the left. The output of the fluorescence detector is sent to a laptop computer via RS232.

To perform fluorescence measurements, the microreactors were placed in the mTFR instrument (see Figure 3) and allowed to equilibrate to 39 °C. Then, measurements were taken each second for a total duration of 60 s. For absolute fluorescence measurements, the first value of each time-series was used whereas drift values were calculated by taking the difference between the first and last measurements.

### 2.3. Mobile Temperature-Controlled Fluorescence Reader (mTFR) Instrument

A combined fluorescence detector/heater instrument (see Figure 3) was developed to perform rtRPA. Heating resistors are used for precise temperature control of the 3D printed microfluidic device while fluorescence is measured by a compact epi-fluorescence detector. The overall dimensions of the instrument are 170 mm × 82 mm × 200 mm.

RPA reaction temperature is maintained by closed-loop proportional–integral–derivative (PID) control. The control loop consists of a CAL 3200 PID temperature controller (CAL Controls, Hertfordshire, UK) that drives four heating resistors as well as a PT-100 temperature sensor. The heating resistors are wired in series and are attached to an aluminum plate that is in contact with the microfluidic device during operation. PID parameters were found by the controller’s automatic tuning function. Incubation temperature is continuously displayed and easily set by front panel controls. 

The sample is located below the excitation/detection window of a highly compact epi-fluorescence detector (ESElog, Qiagen Lake Constance GmbH, Stockach, Germany). The detector has two excitation (E1: 470 nm, E2: 520 nm) and two detection (D1: 550 nm, D2: 600 nm) channels allowing for the simultaneous measurement of two fluorescent dyes. The output of the detector is directly sent to a laptop computer via RS232 for display and recording. For the detection of rtRPA reactions, the combinations of excitation channel 1 and detection channel 1 (E1D1) and excitation channel 2 with detection channel 2 (E2D2) are used in this work.

### 2.4. DNA Standards

#### 2.4.1. *Klebsiella Pneumoniae* Genomic DNA

A plasmid-free *Klebsiella pneumoniae* isolate was grown in LB media overnight at 28 °C in an orbital shaker at 180 rpm. Genomic DNA was isolated using the QIAmp DNA Blood Mini kit (Qiagen, Hilden, Germany) and quantified by PicoGreen assay. The DNA was subsequently digested by the restriction enzyme *Eco*RI at 37 °C for 15 min followed by enzyme inactivation at 65 °C for 20 min. Finally, a dilution series from 10^6^ down to 10^2^ genome copies/µL was prepared.

#### 2.4.2. *bla*_NDM-1_ Resistance Gene

A *Klebsiella pneumoniae* isolate carrying plasmid-encoded *bla*_NDM-1_ was acquired from the National Reference Laboratory for multidrug-resistant gram-negative bacteria for Germany (Ruhr-University Bochum) and kept on LB agar. For liquid culture, bacteria were grown overnight at 28 °C in an orbital shaker at 180 rpm. Plasmids were isolated from liquid culture using the QIAprep Miniprep kit (Qiagen, Hilden, Germany). The *bla*_NDM-1_ gene was then amplified by PCR with forward primer 5′-CCAATATTATGCACCCGGTCGC and reverse primer 5′-ATGCGGGCCG TATGAGTGATT. The resulting amplicon was ligated into a pCRII vector using a TA cloning kit (Invitrogen, Carlsbad, CA, USA). TOP10F’ chemically competent cells (Invitrogen, Carlsbad, CA, USA) were transformed with the ligation product and clones carrying the *bla*_NDM-1_ insert were selected by blue/white screening. Clones were grown in LB medium overnight at 37 °C in an orbital shaker at 180 rpm and plasmids were isolated using the QIAprep Miniprep kit. The correct sequence of the *bla*_NDM-1_ insert was confirmed by Sanger sequencing. The final *bla*_NDM-1_ standard was then prepared by measuring plasmid concentration using the PicoGreen assay (Thermo Fisher, Waltham, MA, USA) on a NanoDrop 3300 instrument and preparation of a dilution series containing 10^6^ down to 10^2^ plasmid copies/µL.

### 2.5. Recombinase Polymerase Amplification (RPA)

#### 2.5.1. Reaction Setup

**Figure 4:**
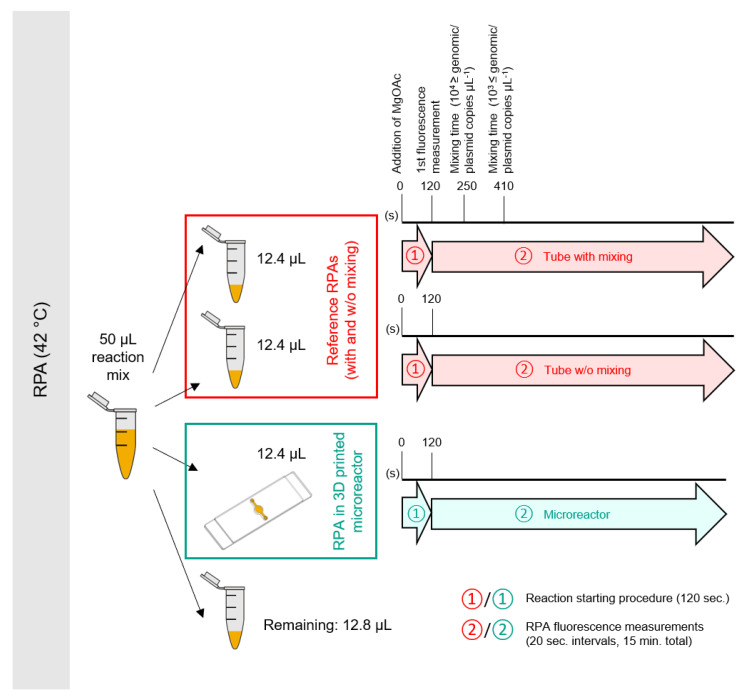
Distribution and incubation/mixing scheme for rtRPA reactions.

Recombinase polymerase amplification (RPA) was performed using reagents supplied with the TwistAmp exo kit (TwistDx, Cambridge, UK). For each singleplex (duplex) reaction, a rehydration mix consisting of 12.2 µL (11.6 µL) PCR-grade water, 29.5 µL (29.5 µL) rehydration buffer, 2.1 µL (1.05 µL) of each primer, and 0.6 µL (0.6 µL) exo probe (10 µM stocks) was prepared and used to rehydrate a freeze-dried reaction pellet. Template DNA (1 µL) was then added to each reaction and 2.5 µL of a 280 mM magnesium acetate (Mg(OAc)_2_) solution was pipetted into the lid of each tube. In the case of negative control reactions, the DNA template was replaced by 1 µL of PCR-grade water. To initiate the reactions, the tubes were briefly spun to add the Mg(OAc)_2_ solution to the reaction mix. The tubes were then vortexed and spun again to collect the reagents at the bottom. The activated reaction mix was quickly distributed to new tubes and the 3D printed microreactor according to the scheme detailed in Figure 4 and immediately placed into an ESEQuant (Qiagen Lake Constance GmbH, Stockach, Germany) isothermal fluorescence reader for the tubes and the mTFR instrument for the 3D printed microreactor. Both devices internally use the same fluorescence detector (ESElog, Qiagen Lake Constance GmbH, Stockach, Germany). Since mixing of the RPA reaction a few minutes after the reaction has been initiated has been shown to improve amplification performance, we divide the control reaction into one that is mixed and one that is not mixed for comparison to the microreactor. For reactions that are mixed, tubes are removed from the ESEQuant device at the times indicated in Figure 4 vortexed for a few seconds, spun down in a tabletop centrifuge and immediately placed back into the ESEQuant. 

For positive control reactions, a rehydration mix (47.5 µL) consisting of 29.5 µL rehydration buffer, 10 µL of a 1/10 dilution of positive control DNA, and 8 µL positive control primer/probe mix was used to rehydrate a freeze-dried reagent pellet. The rehydrated reaction mix was then used as detailed previously with the exception that no control reaction with mixing was performed.

#### 2.5.2. RPA Primers and exo Probes

After initial tests with a previously published rtRPA assay [14] targeting *khe* [35] (AF293352) yielded unsatisfactory results in our hands, we constructed a new assay using the primedRPA [36] primer/probe design software. Both primers, as well as the exo probe, were checked for specificity by BLAST alignment against representative *Enterobacteriaceae* genomes. A further primer screen was also carried out with additional manually designed primer pairs, however, none gave a better performance than the pair generated by primedRPA (data not shown). The *khe* exo probe is labeled with FAM and detected in channel E1D1.

The rtRPA assay targeting *bla*_NDM-1_ (NC_023908.1, region 107945…108861) was constructed by manual selection of suitable priming and probe regions according to the manufacturer’s suggestions [37] and selectivity was checked by BLAST alignment to representative sequences. A primer screen was then carried out to select the primer pair with the best amplification performance and lowest limit of detection. Assay specificity was then confirmed by a negative panel containing DNA extracts from 27 bacterial and fungal species as well as human DNA. Further details on the development of the *bla*_NDM-1_ assay is available from the authors upon reasonable request. The *bla*_NDM-1_ exo probe is labeled with LightCycler Red 610 (LC610) and detected in channel E2D2.

Table 3 gives an overview of the rtRPA primers and probes used in this work.

### 2.6. Fluorescence Data Processing

For rtRPA measurements, raw fluorescence data was background corrected by first finding the lowest value of each time-series which was then subtracted from all data points in the same time series. All nonzero data points taken before the lowest value were then set to zero to establish a stable baseline. This background corrected data was then fitted to either a four- (l4) or five-parameter (l5) logarithmic model using the R language *qpcR* package [38]. The time-to-positive (TTP) value was then determined from the fitted data by thresholding. Thresholds were obtained by first calculating the mean and standard deviation (stdev) of each no template control (NTC) reaction from 560 s to 1000 s and then defined as mean + four standard deviations. In case of multiple NTC reactions, the highest value was chosen.

## 3. Results and Discussion

### 3.1. Influence of Post Curing on Autofluorescence

**Figure 5:**
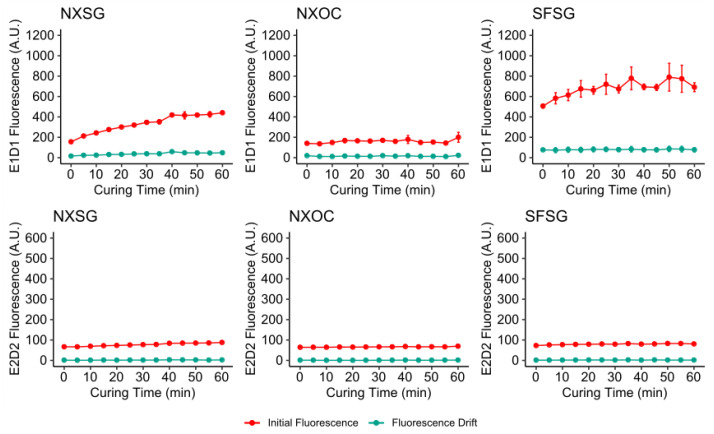
Results of background fluorescence and fluorescence drift measurements for microreactors post cured at room temperature. All measurements were performed on three microreactors for each material.

**Figure 6:**
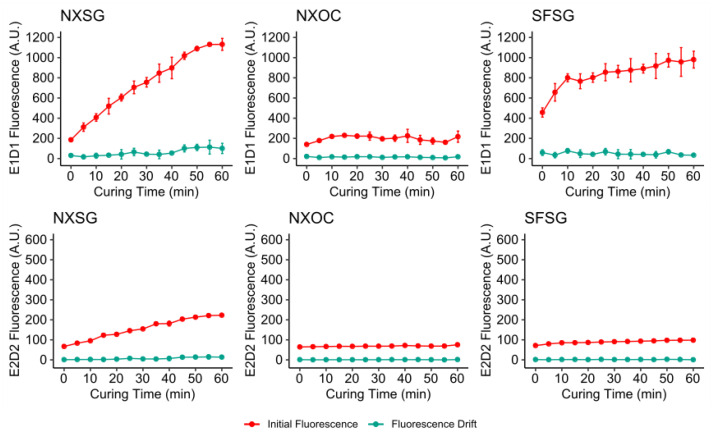
Results of background fluorescence and drift measurements for microreactors post cured at 60 °C. All measurements were performed on three microreactors for each material.

Figure 5 and Figure 6 show the autofluorescence and fluorescence drift measured for increasing curing durations at room temperature and 60 °C. It can be observed that both parameters are highly dependent on both the printing material and curing temperature as well as excitation/detection wavelength with higher wavelengths displaying lower variation in fluorescence. 

NXSG and SFSG display a significant increase in E1D1 during prolonged curing, whereas E2D2 fluorescence increases only minimally. In contrast, the background fluorescence and drift for NXOC are almost independent of curing duration in both fluorescence channels.

Curing at 60 °C magnifies the increases in background fluorescence that were observed for curing at room temperature. Again, NXSG and SFSG show strong correlation of rising fluorescence levels with extended curing for E1D1. Additionally, E2D2 shows a larger increase for NXSG and a slight rise for SFSG in comparison to curing at room temperature. For NXOC, E1D1 fluorescence for curing at 60 °C is more variable, but no significant change is observable. For E2D2, NXOC fluorescence is almost identical to that observed for curing at room temperature.

In conclusion, curing at elevated temperatures should be avoided and curing times should be kept as low as possible if low autofluorescence and fluorescence drift are desired. NXOC is seen as the most suitable printing material in this regard, as it shows very low autofluorescence in both channels that is almost independent of post curing duration. 

### 3.2. Influence of Aging on Autofluorescence

**Figure 7:**
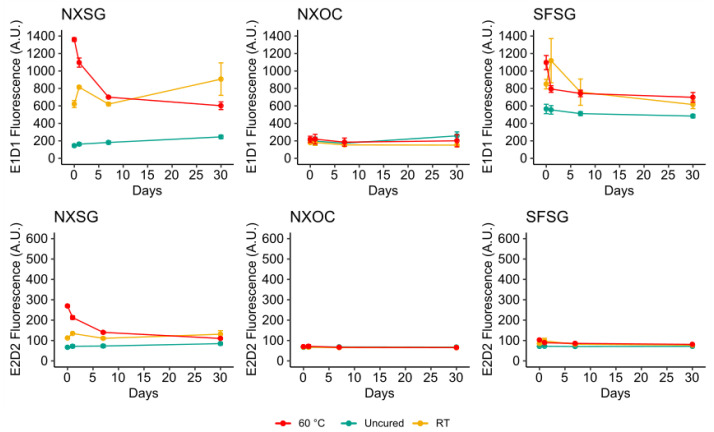
Influence of aging on the background fluorescence of microreactors. All measurements were performed on three microreactors for each material and post curing protocol.

Microreactors were let to age at ambient laboratory conditions. Fluorescence measurements were taken at the day of printing and post curing, after one and seven days and after 30 days. The results of these measurements are presented in Figure 7. Both NXSG and SFSG show strong initial E1D1 autofluorescence for post curing performed at room temperature and 60 °C which decreases (60 °C) or first increases and then decays (room temperature) with longer storage time. For uncured microreactors, lower autofluorescence is observed that displays a less pronounced change over time. For E2D2, only NXSG displays high initial autofluorescence for curing at 60 °C which decays with longer storage to match that of both the uncured microreactors and those post cured at room temperature. In contrast, NXOC does not show large variations in autofluorescence for any of the tested post curing protocols or storage durations. 

### 3.3. Influence of 3D Printing Materials on RPA Kinetics

**Figure 8:**
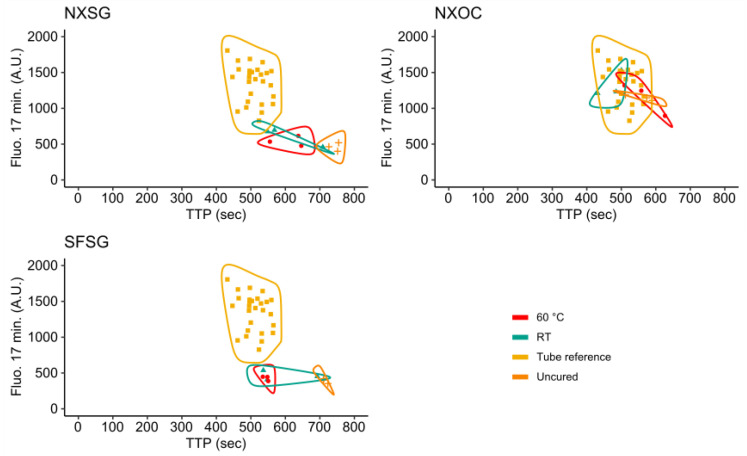
Scatterplot of the time to positive (TTP) value against the final background corrected fluorescence after 17 min for all investigated 3D printing materials and post curing conditions.

Positive control rtRPA reactions were performed in microreactors printed from NXSG, NXOC, and SFSG according to the protocol detailed in Section 2.5.1. Reaction setup. Figure 8 shows a scatter plot of the time-to-positive (TTP) values versus final fluorescence 17 min (2 min reaction setup + 15 min signal acquisition) after Mg(OAc)_2_ addition for each tested material and post curing protocol. Also shown are the data points obtained for reference measurements in microcentrifuge tubes.

The ideal measurement would appear in the top left of the plotting plane with a combination of a low TTP value and high final fluorescence. Additionally, data points obtained from 3D printed microreactors should cluster together with data points of the microcentrifuge tube reference measurements.

For both NXSG and SFSG, all data points are located outside and to the lower right of the tube reference cluster indicating measurement results that are of lower performance than the references. Independent of the post curing protocol, almost all data points obtained for reactions performed in microreactors printed from NXOC cluster well with the reference measurements. Of these values, those obtained after post curing for 60 min at room temperature yielded the lowest TTP values in combination with the highest final fluorescence. However, all three post curing protocols lead to comparable results for microreactors printed from NXOC, indicating that post curing may not even be necessary for this material.

### 3.4. RPA of the khe and bla_NDM-1_ Genes

#### 3.4.1. Singleplex Assay

**Figure 9:**
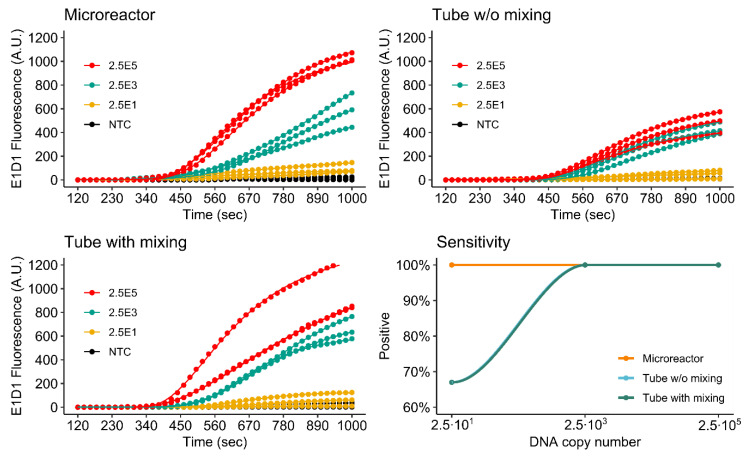
Detection of *khe* by singleplex rtRPA.

**Figure 10:**
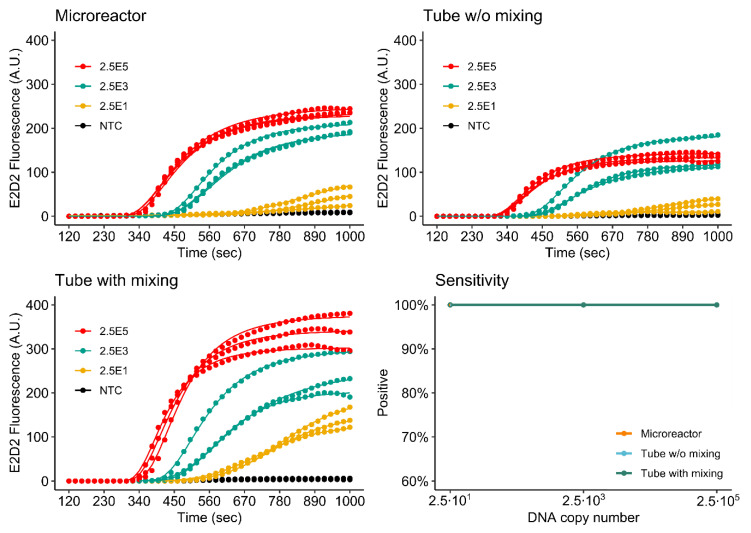
Detection of *bla*_NDM-1_ by singleplex rtRPA.

Figure 9 and Figure 10 show the detection of both the *khe* and *bla*_NDM-1_ genes in microreactors printed from NXOC as well as reference measurements in microcentrifuge tubes with- and without mixing during amplification. 

For the *khe* gene, (FAM, E1D1) all tested dilutions are detected by the assay performed in the microreactor. Final fluorescence intensities measured for the microreactor are close to those measured for the reference assay with mixing. The reference without mixing displays significantly lower final fluorescence. This is an interesting result, as the reagents inside the microreactor are not mixed during amplification, indicating that mixing is of higher advantage if the assay is performed in microcentrifuge tubes. For microfluidic applications, this finding is of importance as mixing is difficult to perform once the RPA reagents are inside a microreactor.

As for *khe*, all tested dilutions of *bla*_NDM-1_ (LC610, E2D2) are detected by the assay performed in the microreactor. In this case however, final fluorescence values measured in the microreactor are lower than those measured for the mixed reference, but higher than the reference without mixing.

These results also show that there is an advantage to performing RPA in a microfluidic setup, as almost the same assay performance is achieved without the need for mixing when compared to the results obtained for the mixed microcentrifuge tube assay. A likely explanation for the observed improved performance may be found in the flat geometry of the microreactor. Due to the high viscosity of the RPA reagents, initial amplification occurs in discrete microscopic zones around each target molecule. We hypothesize that due to the chamber geometry and optical setup, more of these amplification zones are in the optical focus of the epi-fluorescence detector. For the tube assay, RPA reagents have a higher distribution in the vertical axis and are less optically accessible to the fluorescence detector. The ESEquant device used for the reference measurements uses an ESElog epi-fluorescence detector that observes microcentrifuge tubes from the bottom.

#### 3.4.2. Duplex Assay

**Figure 11:**
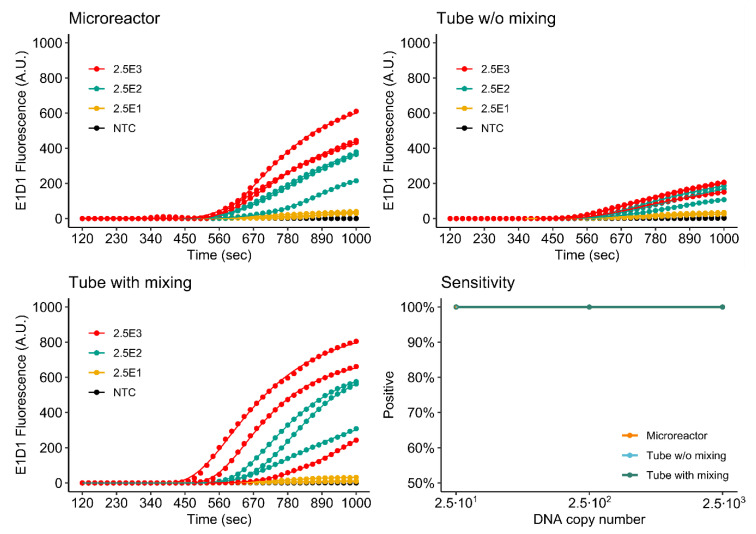
Detection of *khe* by duplex rtRPA.

**Figure 12:**
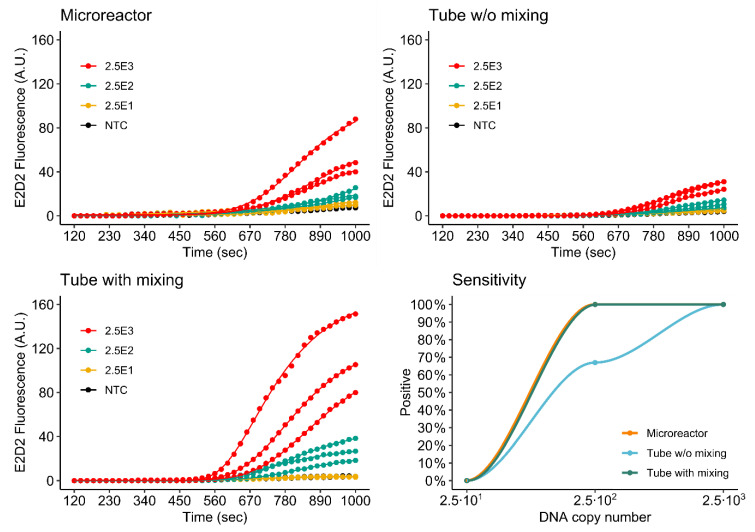
Detection of *bla*_NDM-1_ by duplex rtRPA.

Figure 11 and Figure 12 show the simultaneous detection of *khe* and *bla*_NDM-1_ by duplex rtRPA in the 3D printed microreactor as well as the mixed and non-mixed reference reactions. As is to be expected because of the halved primer concentrations, the duplex assay shows reduced fluorescence evolution in comparison to the singleplex assays. Again, signal performance of the microreactor is observed to be better than that of the non-mixed control. For *khe*, detection sensitivity is identical to that of the singleplex assay. For *bla*_NDM-1_, sensitivity is decreased by one order of magnitude to 2.5 × 10^2^ DNA molecules for the microreactor.

In future work, increased performance may be achieved by further optimization of the multiplex assay, for example, as performed by Kim et al. [12].

#### 3.4.3. Comparison of Single- and Duplex Assays

**Figure 13:**
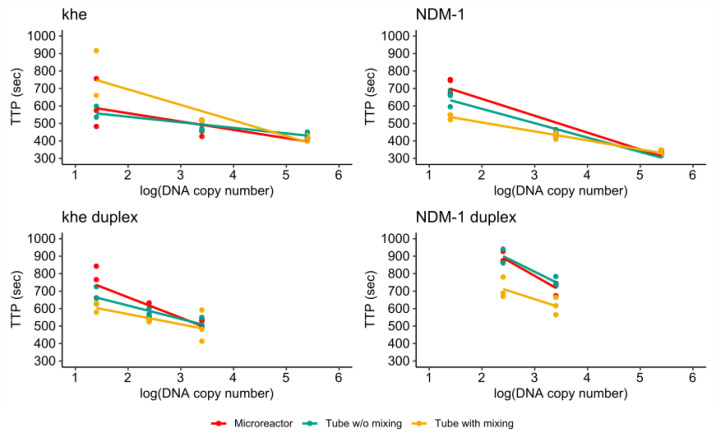
Comparison of TTP-values measured for single- and duplex assays targeting *khe* and *bla*_NDM-1_.

Figure 13 shows the TTP values measured for all single- and duplex assays plotted against the logarithm of the target DNA copy number. For the singleplex assays all values obtained for the two highest target concentrations cluster well together while a higher spread was observed for the lowest concentration. Furthermore, it can be observed that the values obtained for the microreactor and non-mixed control cluster together for all investigated target copy numbers.

For the duplex assay, a higher spread is observed for all measurements. Again, the values obtained for the microreactor and non-mixed control cluster together, and in case of the *bla*_NDM-1_ duplex assay are distinct from those obtained for the mixed reference.

These results indicate that rtRPA reactions in microreactors can deliver similar performance as assays in microcentrifuge tubes. Of high importance is the observation that the advantageous effect of mixing is assay dependent with mixing improving assay performance for the singleplex detection of *bla*_NDM-1_ at low copy numbers, while the detection of *khe* is not improved. For the duplex assay, mixing improves detection performance for both genes. However, overall performance of the microreactor assay is comparable and may be improved by further optimization of the primer/probe concentrations.

## 4. Summary and Conclusions

In this work we expanded the state-of-the-art by demonstrating a DLP 3D printing approach that is suitable for the manufacture of microfluidic devices for real-time RPA. First, we investigated three commercially available biocompatible printing materials, normally used for dental applications, for their autofluorescence and fluorescence drift as well as the effect of aging in dependence on different post curing protocols. We found that devices printed from NXOC that were subjected to either no post curing or post curing at ambient temperature displayed very low autofluorescence and autofluorescence drift that were not strongly influenced by aging. In the next step, we investigated the performance of an rtRPA positive control assay when performed inside 3D printed microreactors. Final fluorescence after 17 min incubation and the time to positive (TTP) value were used as evaluation metrics. Again, devices printed from NXOC followed by either no post curing or post curing at ambient temperature showed the best performance. Based on these results, we demonstrated the amplification of the *khe* and *bla*_NDM-1_ genes inside 3D printed microreactors and compared assay performance to non-mixed and mixed controls both for singleplex as well as a duplex assay. For the singleplex assays, we achieved a detection limit (at 100% detection) of 2.5 × 10^1^ DNA copies. When both assays were combined into a duplex assay, we achieved a limit of detection of 2.5 × 10^1^ copies of *khe* and 2.5 × 10^2^ copies of *bla*_NDM-1_.

In conclusion, our work demonstrates the compatibility of real-time RPA with devices manufactured by DLP based 3D printing. We were able to identify an optically clear 3D printing material that allows for the use of amplification detection by the very popular and inexpensive FAM labeling. This result was quite unexpected, as the DLP 3D printing process uses resins that contain chemicals (such as UV blockers) that are fluorescent in the same wavelength ranges. Additionally, we demonstrated that by choosing a longer detection wavelength, even lower autofluorescence could be achieved. However, longer wavelength fluorophores tend to have lower quantum yields necessitating more sensitive detection equipment. 

A further unexpected result was that for NXSG and SFSG autofluorescence increased with extended light exposure during post curing. This finding contrasts our expectation that longer post curing reduces autofluorescence by bleaching of the responsible fluorophore inside the material. Additionally, autofluorescence was further increased when post curing was carried out at elevated temperature (60 °C). These findings show that, counterintuitively, as little post curing as possible should be performed if low autofluorescence is desired.

As has been shown in other work, RPA reagents are readily compatible with high surface to volume ratio reaction vessels without the need for surface passivation by e.g., BSA or PEG. It is likely that due to the high amounts of PEG and proteins present in the RPA reaction mix, RPA “self-passivates” its reaction vessel.

In future work, with the addition of suitable sample preparation technology, fully monolithic micro total analysis systems (µTAS) for nucleic acid detection may be manufactured just by 3D printing and pre-loading of (lyophilized) reagents. Such devices have a multitude of applications in diverse areas such as the medical field, food and water analysis or plant pathogens.

## Figures and Tables

**Table 1:** 3D printing materials used in this study.

Material	Manufacturer	Abbreviation
NextDent Surgical Guide	NextDent B.V., Soesterberg, The Netherlands	NXSG
NextDent Ortho Clear	NextDent B.V., Soesterberg, The Netherlands	NXOC
SolFlex Surgical Guide	VOCO GmbH, Cuxhaven, Germany	SFSG

**Table 2:** Details of the three post curing protocols used for this work.

Protocol	Illumination (min)	Temperature
Uncured	-	-
RT	60	Ambient (~23 °C)
60 °C	60	60 °C

**Table 3:** Recombinase polymerase amplification (RPA) primers and exo probes used in this work.

Name	Sequence 5′-3′
*khe* Forward	ACACTTTTCTCAATAACACCGAGCAGGAGGTTC
*khe* Reverse	CGCATAGTGCGCCGCGCTTCGCCCCTTCCCCGG
*khe* exo probe	CGCTCAATCCAGGCTATGCCGCGACGCGCCAGGA(dT-BHQ1)C(dspacer)(dT-FAM)TGGGTTGACCATCC-PH
*bla*_NDM-1_ Forward	GACCAGACCGCCCAGATCCTCAACTGGATCAAGCA
*bla*_NDM-1_ Reverse	CTGGTTCGACAACGCATTGGCATAAGTCGCAA
*bla*_NDM-1_ exo Probe	CCCCGCCGCATGCAGCGCGTCCATACCGCCCA(dT-BHQ2)(dspacer)(dT-LC610)TGTCCTGATGCGCG-PH

## Abbreviations

(rt)RPA	(real-time)Recombinase Polymerase Amplification
mTFR	Mobile temperature-controlled fluorescence reader
NXSG	NextDent Surgical Guide
SFSG	SolFlex Surgical Guide
NXOC	NextDent Ortho Clear
BSA	Bovine Serum Albumin
PEG	Polyethylene glycol
TTP	Time-to-positive
DLP	Digital Light Processing
PID	Proportional-integral-derivative
IPA	Isopropyl alcohol
SLA	Stereolithography
FDM	Fused Deposition Modeling
*khe*	*Klebsiella* haemolysin
*bla*_NDM-1_	New Delhi metallo-β-lactamase 1
NTC	No template control
AMR	Antimicrobial Resistance
FAM	Carboxyfluorescein
LC610	LightCycler Red 610
KPN	*Klebsiella pneumoniae*
HTR	Horizontal Gene Transfer
UTI	Urinary Tract Infection
µTAS	Micro Total Analysis Systems
Mg(OAc)_2_	Magnesium acetate
